# Investigation of Lipid Transfer between Model Membranes and the Effect of a Transport Protein on Trafficking

**DOI:** 10.1021/acsomega.5c07525

**Published:** 2025-10-29

**Authors:** Evelyn W. Cheng, Megan McDonald, Suli Kamholtz-Roberts, Virginia Durcan, Ashlee M. Plummer-Medeiros

**Affiliations:** Bryn Mawr College, Chemistry Department, 101 N Merion Ave, Bryn Mawr, Pennsylvania 19010, United States

## Abstract

The phospholipid composition of biological membranes is precisely controlled, and slight tuning of lipid content can have drastic effects on macroscopic membrane properties and the critically important functions of membrane-associated proteins. Both spontaneous and protein-catalyzed exchange of lipids between membranes are mechanisms for modulating membrane lipid composition. Here, an *in vitro* platform for the analysis of the transfer of tagged lipid derivatives between synthetic liposomes is developed and used to systematically investigate the effect of bulk solution properties, such as temperature, pH, and liposome concentration, on the exchange of lipids between model membranes. The impact of a representative lipid transport protein was studied to understand the role of protein concentration and membrane anchoring on lipid exchange. The spontaneous exchange of lipids between model membranes is enhanced by the addition of the model transport protein when protein–membrane interactions are mediated through anchoring. These data suggest that membrane-binding proteins may play an integral role in catalyzing the transfer of lipids between membranes.

## Graphical abstract

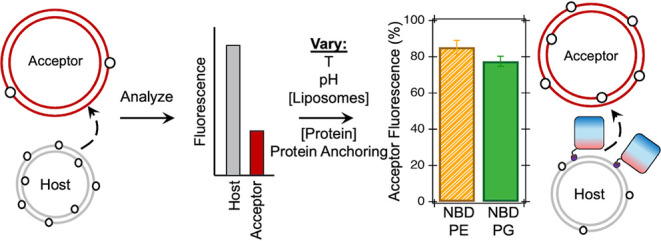

## Introduction

All cells are encapsulated by a protective membrane barrier, which contains two sandwiched layers of phospholipids. Membranes contain many different combinations of phospholipids with variable acyl chains and polar headgroups and this finely tuned lipid composition is critical for the biochemical functionality of the membrane.^1^ Accordingly, dysregulation or changes to this dynamic lipid content are hallmarks of various diseases including cancer and atherosclerosis.^2,3^ Physiological membranes have precise compositions that dictate their macroscopic behavior,^4,5^ such as physical phase, as bilayers can undergo thermally induced transitions between various phases that depend on their composition.^6,7^ Also, varying lipid headgroups have distinct polar or ionizable functional groups—their protonation states dictate their intermolecular interactions with other lipids and membrane-binding macromolecules.^8^ Assessing how bulk solution conditions, such as temperature and pH, impact lipid behavior is critical to understanding the properties of membranes.

In addition to phospholipids, biological membranes include other macromolecules, such as membrane-bound or membrane-embedded proteins, which perform a variety of functions that are required for cell survival. For instance, peripheral membrane proteins bind to membrane surfaces via complex electrostatic and hydrophobic interfaces and dysregulation of these reversible interactions plays a critical role in the development of diseases, including cancer.^9^ Integral membrane proteins span biological membranes and their critical functions are finely tuned by their surrounding local lipid environment.^10^ Membrane phospholipid composition and its biophysical properties directly affect the activities of both peripheral and integral membrane proteins.

Dynamic changes in membrane lipid content modulate lipid–lipid and lipid–protein interactions—the exchange of lipids between biological membranes often facilitates these compositional changes. This transfer and the subsequent delicate balance of lipid content between cell membranes are commonly mediated by lipid transport proteins.^11^ The role of these transport proteins at eukaryotic membrane-contact sites, or regions where two membranes are physically anchored by a tethering protein, was recently identified as a novel therapeutic target for cancer drug development.^12,13^ The exchange of lipids has also been reported to occur in the absence of proteins, albeit slowly,^14,15^ and in simpler organisms. Gram-negative bacteria, like*Escherichia coli*, are surrounded by a double membrane barrier and several lipid transfer proteins have been implicated in facilitating the movement of bacterial lipids between these membranes.^16–19^ Here, bacterial membranes are used as a model system to investigate the spontaneous and protein-catalyzed transfer of lipids between membranes.

To interrogate the role of lipid transfer proteins in membrane composition changes, the *E. coli* Lipophilic Envelope-Spanning Tunnel protein B (LetB, previously known as YebT) is used here as a representative transfer protein. This protein was originally identified as a phospholipid-trafficking protein belonging to the Mammalian Cell Entry (MCE) protein family.^20^ LetB contains an N-terminal transmembrane (TM) helix, which anchors the protein to the bacterial inner membrane, followed by seven modular MCE domains (Figure S1A). The structure of LetB lacking its TM anchoring helix (i.e., LetB-ΔTM) revealed that LetB is hexameric; therefore, six MCE domains come together to form a ring with a hydrophobic interior tunnel.^21,22^ The seven stacked MCE rings of the hexameric LetB are approximately 20 nm long, wide enough to span the aqueous space between the bacterial membranes, and physically connect the membranes to shuttle lipids between them, similar to a eukaryotic membrane-contact site (Figure S1B). Inward-facing residues in the LetB hexameric tunnel have been shown to cross-link to phospholipids and LetB copurifies with lipids containing the polar headgroups that are characteristic of bacterial membranes: Phosphatidyl-ethanolamine (PE) and Phosphatidyl-glycerol (PG)^17,21,23^ (Figure S2). Together, previous work on LetB suggests that it is a fitting model lipid transfer protein to introduce into the *in vitro* lipid transfer assay developed herein to better understand how this protein shuttles lipids between model membranes.

This work constructs an experimental framework for the investigation of lipid trafficking between synthetic membrane mimetics and liposomes made of endogenous*E. coli* lipids. This platform is utilized to investigate the spontaneous transfer of bacterial phospholipid analogs between liposomes and the effect of bulk solution properties on their flux. Additionally, the model lipid-trafficking protein, LetB-ΔTM, is incorporated into this *in vitro* lipid transport system to understand how this membrane-binding protein facilitates the trafficking of lipids between liposomes. Based on previous studies, it was hypothesized that LetB-ΔTM can efficiently shuttle both types of endogenous bacterial lipids between model membranes and overcome the kinetic barrier associated with spontaneous lipid transfer.

## Results and Discussion

### Characterization of Spontaneous Lipid Exchange

To assess the spontaneous exchange of lipids between liposomes, the transfer of acyl-chain fluorescently tagged lipids (e.g., 7-nitro-2-1,3-benzoxadiazol-4-yl-amino or NBD-tagged PE and NBD-PG) was measured under a variety of conditions. Host or donor liposomes begin with 1% tagged lipids, while acceptor liposomes contain only*E. coli* polar extract; these liposomes were prepared with diameters of 100 and 400 nm, respectively, with sizes confirmed by Dynamic Light Scattering (DLS, Figure S3). After mixing and an incubation period, the host and acceptor liposomes are separated and the fluorescence of each is measured (Figure 1).^24^ Data shown in Figure 1 indicate measured lipid transfer over a time course of 30 min; henceforth, data shown typically utilize a 30 min time point, as this is representative of lipid transfers measured herein. A plateau in the flux of both NBD-PE and NBD-PG after 24 h is observed, which is consistent with previously reported half-times for the slow spontaneous transfer of lipids between liposomes composed of endogenous*E. coli* lipids^25^ (Figure S2).

**Figure 1:**
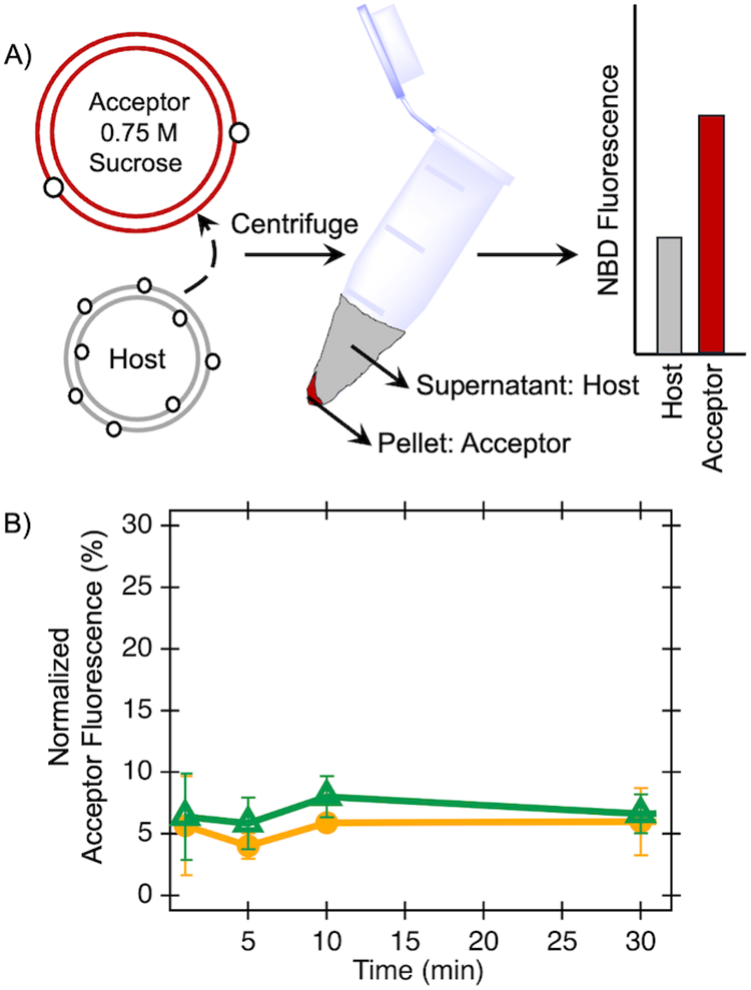
Transfer of NBD-tagged lipids (open circles) between host (gray) and acceptor (red) liposomes is measured following incubation of liposome mixtures, centrifugation, and subsequent separation (**A**). The NBD fluorescence compositions of host and resuspended acceptor liposomes are measured. NBD-tagged PE (orange) and PG (green) lipids are tested independently and kinetic experiments monitor the transfer of lipids over a time course (**B**). Experiments here include 400 μM mixtures of host liposomes with 100 nm diameters and acceptor liposomes with 400 nm diameters containing 0.75 M sucrose. Averages of 3 biological replicates with standard deviations are shown.

The microscopic and macroscopic behavior of lipids and membranes depends on thermal energy, as bilayers are known to undergo a gel-to-liquid crystalline phase transition at characteristic temperatures. The*E. coli* polar lipids utilized here have a phase transition temperature of ∼2 °C.^26^ Below the phase transition, lipids exhibit diminished motion and have lower mobility.^25^ Measurements of lipid exchange at temperatures ranging from 4 to 37 °C consistently show ∼5% to 10% transfer of both lipid types, except for NBD-PG transfer at 37 °C, which exceeds 15% (Figure 2A). Overall, the data shown in Figure 2A suggest that the transfer of lipids between liposomes in this tested temperature range is marginally impacted. The increased transfer of NBD-PG observed at 37 °C may arise from thermal energy assisting in overcoming the enthalpic cost of lipid transfer between liposomes, although this has been particularly notable in transfer studies of lipids with varying acyl chain lengths.^14^ Tagged lipids here have identical acyl chain lengths and therefore likely exhibit minimal transfer temperature sensitivity.

**Figure 2:**
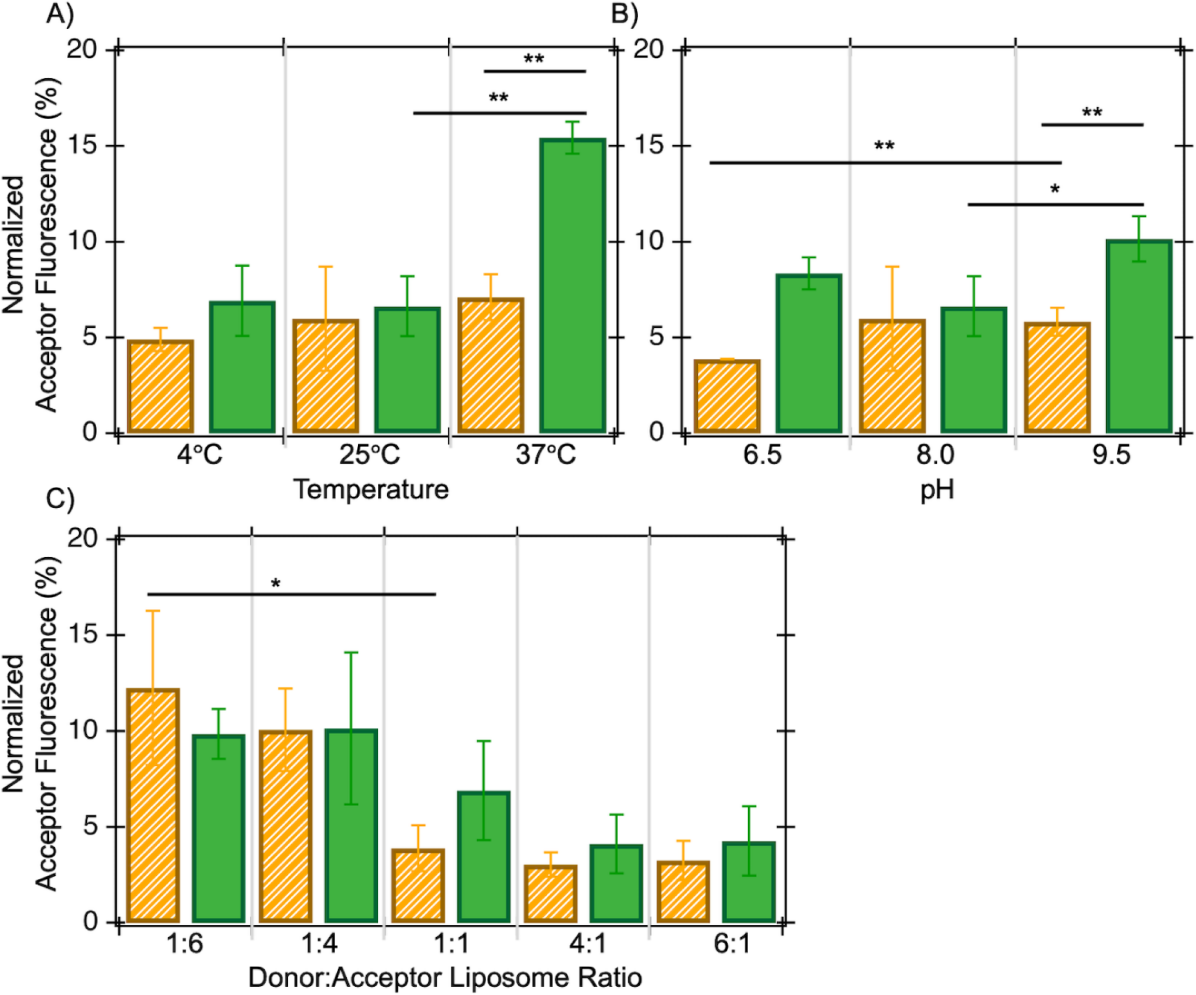
Transfer of NBD-tagged lipids between host and acceptor liposomes is measured as a function of temperature (**A**), solution pH (**B**), and varying donor:acceptor liposome molar ratios (**C**). Transfers of NBD-PE and NBD-PG are colored orange and green, respectively. Experiments here include 400 μM mixtures of host liposomes with a 100 nm diameter and acceptor liposomes with a 400 nm diameter containing 0.75 M sucrose separated after 30 min (**A** and **B**). For **C**, the 1:1 molar ratio mixture includes 400 μM mixtures of host liposomes with a 100 nm diameter and acceptor liposomes with a 400 nm diameter containing 0.75 M sucrose separated after 30 min; molar ratios for other mixtures are scaled relative to this. Averages of 3 biological replicates with standard deviations are shown. ** indicates *p* < 0.01 using a 2-tailed *t*-test assuming equal variance; * indicates *p* < 0.05 using a 2-tailed *t*-test assuming equal variance.

Additionally, the effect of pH on the spontaneous transfer of NBD-PE and NBD-PG lipids was quantified (Figure 2B). Under physiological conditions, the PE headgroup is zwitterionic (p*K*_a_ ∼ 9.6), while the PG headgroup has a net negative charge. A slight increase in the transfer of both lipids at higher tested pHs was observed, indicating a modest change to ionization states and corresponding transfer rates in the tested pH range. Similar data were observed for liposome mixtures tested at a different concentration under this pH range (Figure S4). In conclusion, the pH has a minor effect on the spontaneous transfer of NBD-tagged lipids under these tested conditions.

Varying molecular mechanisms exist for spontaneous lipid exchange between membranes: lipids can be transferred through direct physical collisions of liposomes or through the desorption of individual lipid monomers from host liposomes, followed by insertion into acceptor liposomes (Figure S5). To differentiate between these transfer mechanisms, the rate of exchange can be tested for various ratios of host and acceptor liposomes. If a collision-based mechanism is prevalent, the rate of transfer is directly proportional to the concentration of acceptor vesicles.^27^ Previous studies have suggested that lipid transfer between liposomes of endogenous*E. coli* lipids does exhibit a dependence on acceptor liposome concentration,^25^ although other NBD-tagged lipids undergo transfer through the monomeric desorption mechanism.^15^ Figure 2C shows the dependence of NBD-PE and NBD-PG transfer on the donor:acceptor liposome ratio. The transfer of PE shows a statistically significant dependence on acceptor liposome concentration, indicating that this transfer likely proceeds through a liposome-collision mechanism. PG transfer follows a similar trend yet these data lack statistical significance.

### Exchange in the Presence of a Lipid Transport Protein

To interrogate the effect of a representative lipid transfer protein, the LetB protein was introduced into the above experiments to determine its effect on the measured transfer rates of NBD-PE and NBD-PG, as these lipid headgroups are endogenous to*E. coli*. Indeed, LetB has been copurified with both lipids, which are likely substrates for transport through the LetB tunnel.^17,21,23^ LetB protein lacking its N-terminal TM and containing a C-terminal poly-Histidine tag (LetB-ΔTM-His_6_) was expressed and purified as previously described^21^ (Figure S1) and incorporated into the *in vitro* lipid transfer assay above at varying lipid:protein molar ratios. Previous studies of protein-coupled lipid transfer have shown increased rates of exchange exceeding 10^6^ ^28^—however, a modest increase in the NBD-PE and NBD-PG transfer in the presence of LetB-ΔTM was observed. Only 10% of the NBD-tagged lipid for both tested headgroups was transferred in the presence of a 100:1 lipid-to-LetB-ΔTM molar ratio (Figure 3A and 3B). This lack of LetB-ΔTM effect may be due to the inherent flexibility of the LetB protein or due to the absence of the LetB N-terminal TM domain.^22,29,30^ SDS-PAGE analyses suggest that LetB-ΔTM does not effectively bind to liposomes under these tested conditions (Figure S6). To better understand the impact of LetB-ΔTM liposome binding, protein-anchoring lipids were incorporated into the model host/acceptor liposomes.

**Figure 3:**
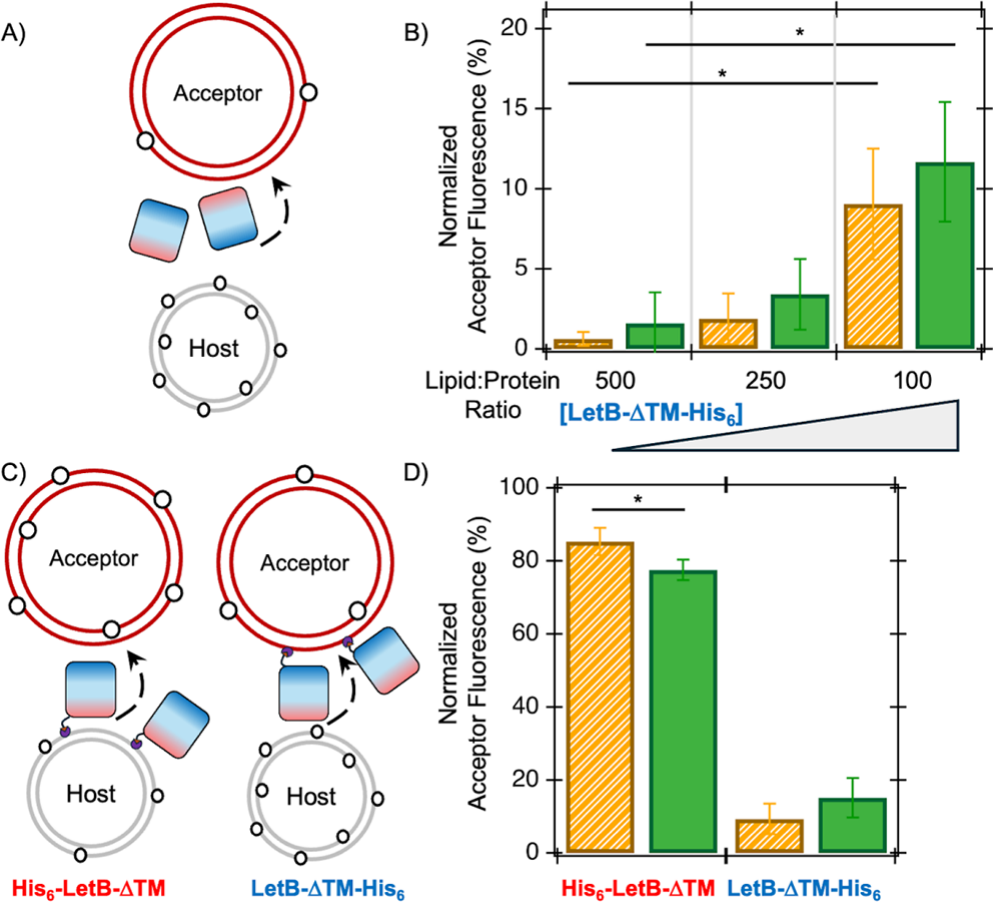
LetB-ΔTM affects the measured transfer of lipids in a dose-dependent manner (**A** and **B**); also, anchoring LetB-ΔTM to host liposomes results in increased lipid transfer, unlike anchoring to acceptor liposomes (**C** and **D**). The transfer of NBD-tagged lipids (open circles) between host (gray) and acceptor (red) liposomes is measured in the presence of LetB-ΔTM; the N- and C-termini of LetB-ΔTM are shown in red and blue, respectively (**A**). Experiments here include 400 μM mixtures of host liposomes with a 100 nm diameter and acceptor liposomes with a 400 nm diameter containing 0.75 M sucrose, with varying molar ratios of LetB-ΔTM-His_6_ added, and separated after 30 min. Background spontaneous transfer is subtracted from the LetB-ΔTM-catalyzed amounts here (**B**). The transfer of NBD-tagged lipids (open circles) between host (gray) and acceptor (red) liposomes is measured in the presence of LetB-ΔTM anchored to host and acceptor liposomes via N- or C-terminal Histidine tags, shown at the red and blue ends, and interactions with DGS-NTA­(Ni^2+^) lipids (purple), respectively (**C**). Experiments here include 400 μM mixtures of host liposomes with a 100 nm diameter and acceptor liposomes with a 400 nm diameter containing 0.75 M sucrose, with a 100:1 lipid:LetB-ΔTM ratio, separated after 30 min. Background spontaneous transfer is subtracted from the LetB-ΔTM-catalyzed amounts here (**D**). Transfers of NBD-PE and NBD-PG are shown in orange and green, respectively (**B** and **D**). Averages of 3 biological replicates with standard deviations are shown. * indicates *p* < 0.05 using a 2-tailed *t*-test assuming equal variance.

### Exchange in the Presence of Liposome-Anchored Lipid Transfer Protein

To replicate the membrane anchoring of LetB-ΔTM, the host and acceptor liposomes were modulated to include lipids with (*N*-(5-amino-1-carboxypentyl)-iminodiacetic acid) succinyl nickel salt (e.g., DGS-NTA­(Ni^2+^)) headgroups—this nickel-chelating headgroup binds with tight affinity to poly-Histidine tags,^31,32^ which are used for LetB-ΔTM purification. Strategic placement of a His_6_-tag at the N-terminus (i.e., host liposome surface, Figure 3B) of LetB-ΔTM permits anchoring of His_6_-LetB-ΔTM to the host liposome (Figure 3). Control experiments have shown that incorporation of DGS-NTA­(Ni^2+^) lipids into liposomes has a minimal impact on the spontaneous transfer of NBD-PE and NBD-PG (Figure S7). However, anchoring of His_6_-LetB-ΔTM to host liposomes significantly increases lipid transfer—almost 80% of tagged PE lipids are transferred in the presence of anchored LetB-ΔTM (Figure 3D). While the transfer of NBD-PG is also drastically increased by anchoring of His_6_-LetB-ΔTM, the facilitated flux of NBD-PE is significantly higher than that of NBD-PG under these conditions. Similar anchoring of LetB-ΔTM-His_6_ to acceptor liposomes does not yield a corresponding increase in lipid transfer (Figures 3D and S8). Control experiments have shown minimal effects on lipid transfer of host and acceptor liposome size and curvature (Figure S9).

Co-sedimentation of LetB-ΔTM to host and acceptor liposomes with varying DGS-NTA­(Ni^2+^) and His_6_-tag placement was assessed by SDS-PAGE (Figures 4A and S10)—surprisingly, His_6_-LetB-ΔTM anchored to host liposomes via His_6_/DGS-NTA­(Ni^2+^) lipid interactions cosediments with acceptor liposomes. These data suggest that anchoring of His_6_-LetB-ΔTM to host liposomes may promote productive binding to acceptor liposomes and subsequent NBD-PE and NBD-PG transfer. Lastly, to investigate the effect of His_6_-LetB-ΔTM binding to acceptor liposomes on the interpretation of LetB-ΔTM-mediated lipid transfer, the above host-anchored His_6_-LetB-ΔTM transfer assay experiments were repeated followed by proteolytic cleavage of LetB-ΔTM prior to host and acceptor liposome separation (Figures 4B and S11). Complete proteolysis of LetB-ΔTM followed by separation of host and acceptor liposomes indicates a significant transfer of NBD-lipids to the acceptor liposomes (Figure 4C). Taken together, these data suggest that host-liposome-anchored His_6_-LetB-ΔTM can bind to acceptor liposomes and mediate the transfer of both NBD-PE and NBD-PG lipids under the tested conditions here. However, acceptor-liposome-anchored LetB-ΔTM-His_6_ does not efficiently associate with host liposomes to facilitate lipid transfer.

**Figure 4:**
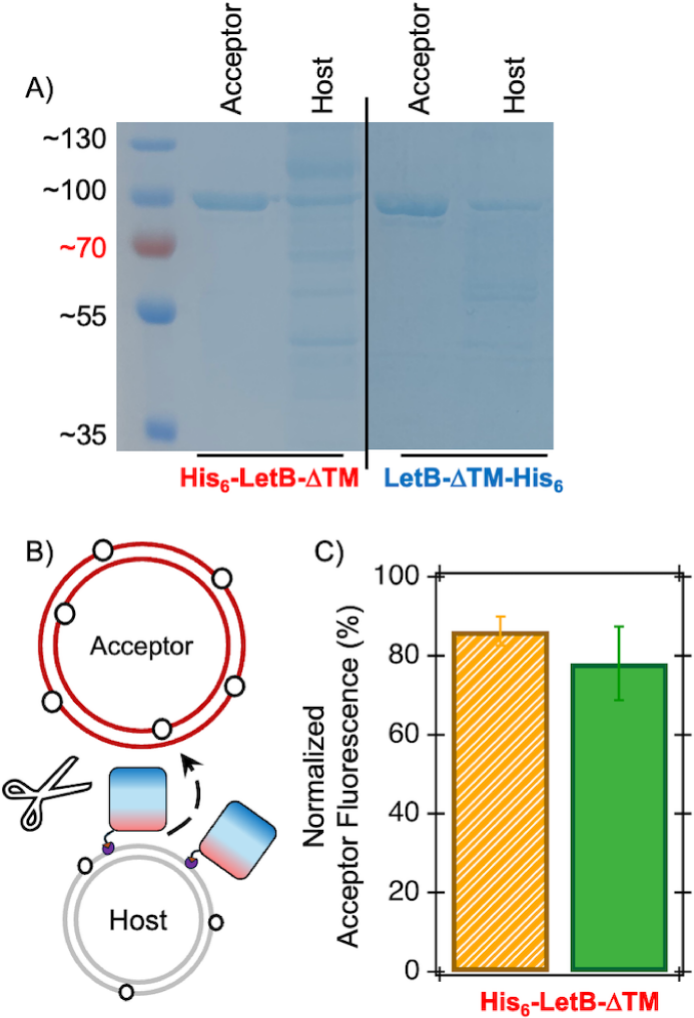
Cosedimentation of LetB-ΔTM with acceptor liposomes occurs for both N- and C-terminally tagged LetB-ΔTM constructs (**A**). Representative SDS-PAGE analysis of separated acceptor and host liposome samples after centrifugation for various tested combinations of donor/acceptor liposomes and His_6_-LetB-ΔTM and LetB-ΔTM-His_6_ (**A**). Experiments here include 400 μM mixtures of host liposomes with a 100 nm diameter and acceptor liposomes with a 400 nm diameter containing 0.75 M sucrose, with a 100:1 lipid:LetB-ΔTM ratio, separated after 30 min, and subjected to SDS-PAGE analysis. The NBD-PE and NBD-PG transfer assay was reproduced with host-liposome-anchored His_6_-LetB-ΔTM, followed by complete proteolytic cleavage of His_6_-LetB-ΔTM protein prior to separation of host and acceptor liposomes for NBD-lipid quantification (**B**). These data suggest that NBD-lipids are transferred to acceptor liposomes prior to centrifugation (**C**). Experiments here include 400 μM mixtures of host liposomes with a 100 nm diameter and acceptor liposomes with a 400 nm diameter containing 0.75 M sucrose, with a 100:1 lipid:LetB-ΔTM ratio. After the 30 min lipid transfer, a 10-fold molar excess of chymotrypsin was added for proteolysis, followed by separation of vesicles. Background spontaneous transfer is subtracted from the LetB-ΔTM-catalyzed amounts here. NBD-tagged PE (orange) and PG (green) lipids are tested independently. Averages of 3 biological replicates with standard deviations are shown.

## Conclusions

The *in vitro* transfer assay described here allows for the characterization of both spontaneous and protein-mediated lipid transfer between synthetic liposomes. Quantification of NBD-PE and NBD-PG transfer between model membranes containing*E. coli*extract demonstrates small amounts of spontaneous flux with a minor dependence on temperature and pH. These data suggest that temperature-mediated phase changes are minimally impacting the observed lipid flux, which is also unaffected by slight changes in headgroup ionization states under these conditions. This exchange of phospholipids is likely mediated through a molecular mechanism involving liposome collision. The utility of this assay is evident through the variety of experimental conditions and mechanistic details that can be obtained through the systematic studies of lipid transfer.

Lipid transfer proteins play an integral role in biological membrane maintenance and this work has investigated how the bacterial MCE protein LetB facilitates lipid exchange between membranes.^11^ Data presented herein suggest that the conditions for protein-catalyzed lipid transfer are critically important, such as the lipid:protein ratio and liposome anchoring. While LetB-ΔTM alone facilitates the transfer of both PE and PG lipids, the flux of lipids to acceptor liposomes is significantly increased when LetB-ΔTM is physically anchored to host liposomes. These findings highlight the importance of LetB-ΔTM anchoring to the bacterial inner membrane-mimicking liposome and suggest a likely role of the LetB N-terminal TM helix in promoting the population of lipid-trafficking-competent conformations of the LetB tunnel, similar to the formation of active membrane-contact sites in eukaryotic cells. It is likely that the molecular mechanism of this protein-mediated lipid flux is distinct from the liposome-collision-based mechanism observed in the absence of protein, and the mechanistic details of protein-catalyzed transport are an exciting avenue of future work. This platform lays the groundwork for illuminating the molecular details of the complex process of lipid exchange at membrane-contact sites, which likely plays an intricate role in various human diseases.^12,13^

## Methods

### Reagents and Chemicals

Liposomes contain*E. coli* Polar Extract (Avanti Polar Lipids, 100600; phospholipid profile of 67% PE, 23.2% PG, 9.8% Cardiolipin) supplemented with 1% 16:0–12:0 NBD-PG (Avanti: 810164P-1 mg; 1-palmitoyl-2-(12-[(7-nitro-2–1,3-benzoxadiazol-4-yl)­amino]­dodecanoyl)-*sn*-glycero-3-[phospho-rac-(1-glycerol)] (ammonium salt)), or 1% 16:0–12:0 NBD-PE (Avanti: 810154P-1 mg; 1-palmitoyl-2-(12-[(7-nitro-2–1,3-benzoxadiazol-4-yl)­amino]­dodecanoyl)-*sn*-glycero-3-phosphoethanolamine), or 5% 16:0 DGS-NTA­(Ni^2+^) (Avanti: 790408C-5 mg; 1,2-dipalmitoyl-*sn*-glycero-3-[(*N*-(5-amino-1-carboxypentyl)­iminodiacetic acid)­succinyl] (nickel salt)). Importantly, NBD-tagged lipids used here have fluorophore tags located on their acyl chains so as not to interfere with headgroup/protein interactions. Typical donor liposomes contain*E. coli* Polar Extract with either 1% 16:0–12:0 NBD-PG or 1% 16:0–12:0 NBD-PE; typical acceptor liposomes contain only*E. coli* Polar Extract.

Tested buffer compositions include: 20 mM Tris (pH 8.0; Sigma Life Sciences, T1503-1 kg), 100 mM NaCl (Sigma-Aldrich, S-3014); 20 mM Potassium phosphate (pH 6.5; Sigma-Aldrich, P3619–1GA), 100 mM NaCl; and 20 mM Glycine (pH 9.5; GoldBio, G-630-1), 100 mM NaCl. Acceptor liposomes were prepared in similar buffers containing 0.75 M sucrose (JT Baker, 4072). All water was purified through a PureLab Flex Elga, Model PF2XXXXM1-US; all buffer stocks were pH-adjusted using a calibrated Fisher Brand AE150 pH meter (13-636-AE153).

pBEL1324 (i.e., plasmid encoding for LetB­(43-877 or ΔTM)-6xHis) was a gift from Gira Bhabha and Damian Ekiert (Addgene plasmid #139874; http://n2t.net/addgene:139874; RRID:Addgene_139874).^21^ The modification of this plasmid to generate His_6_-LetB-ΔTM is described in Supporting Information.

### Lipid Transfer Assays

Lipid films and liposomes are prepared as previously reported.^24,26^ Briefly, 2.5 mg of chloroform-solubilized lipids is aliquoted into glass vials with varying compositions and then dried down under a gentle stream of Ar_*(g)*_ prior to storage at −20 °C. Lipid films are resuspended to be 2.5 mg/mL (∼3480 μM total lipid concentration) prior to sonication (VWR sonicator, Model #93043-960) for 15 min at room temperature followed by vigorous vortexing. Acceptor liposomes are resuspended in buffers containing 0.75 M sucrose. Liposomes are then extruded using a benchtop mini-extruder (Avanti, 610000) with filter supports (Avanti, 610014-1Ea) and either 100 nm Whatman Filter paper (Cytiva, 800309) for donor liposomes or 400 nm Whatman Filter paper (Cytiva, 10417104) for acceptor liposomes. Donor and acceptor liposomes were mixed at varying molar ratios under varying conditions as described above. Following transfer incubations, liposomes were separated by a 30 min centrifugation at 14,000 × rpm (Eppendorf Centrifuge 5418). Donor-liposome-containing supernatants were removed and acceptor-liposome-containing pellets were resuspended in an appropriate volume of buffer. NBD fluorescence of donor and acceptor solutions was then measured using a Tecan Spark fluorescence/absorbance plate reader (λ_exc_ = 460 nm, λ_em_ = 535 nm) in 96-well plates (Fisher, 21-377-203). Donor and acceptor NBD-lipid composition is calculated as a percentage of the measured fluorescence signal of each divided by the total measured signal. Experiments conducted at various temperatures include a preincubation of all buffers and liposomes prior to mixing for at least 1 h (e.g., Fisher Incubator, 150152632).

### LetB-ΔTM Protein Purification

Protein expression, purification, and analysis were completed as previously described^17,21^ (Supplemental Methods). Size-exclusion chromatography and SDS-PAGE confirmed the approximate protein size, and the concentration was determined with Bradford assays.

### Lipid Transfer Assays with LetB-ΔTM

Transfer assays were completed as previously described with a 30 min incubation of 4 μM His_6_-LetB-ΔTM with 400 μM host liposomes containing 1% NBD-PE or 1% NBD-PG with 5% DGS-NTA­(Ni^2+^) and *E. coli* extract extruded to 100 nm in 20 mM Tris (pH 8) and 100 mM NaCl for LetB-host liposome-anchored experiments. For LetB-acceptor liposome-anchored experiments, 4 μM LetB-ΔTM-His_6_ was incubated with 400 μM acceptor liposomes containing 5% DGS-NTA­(Ni^2+^) and *E. coli* extract extruded to 400 nm in 20 mM Tris (pH 8), 100 mM NaCl, and 0.75 M sucrose. 400 μM acceptor or donor liposomes were then added for a 30 min transfer, respectively. Donor and acceptor liposomes were separated by a 30 min centrifugation at 14,000 × rpm. Donor-liposome-containing supernatants were removed and acceptor-liposome-containing pellets were resuspended in an appropriate volume of 20 mM Tris (pH = 8), 100 mM NaCl. NBD fluorescence of donor and acceptor solutions was then measured using a Tecan Spark fluorescence/absorbance plate reader (λ_exc_ = 460 nm, λ_em_ = 535 nm). Donor and acceptor NBD-lipid composition was calculated as a percentage of the measured fluorescence signal of each divided by the total measured signal. Negative control experiments were included with no LetB-ΔTM protein for subtraction of background NBD-PE or NBD-PG transfer.

### SDS-PAGE Analysis

Prior to Sodium Dodecyl Sulfate Polyacrylamide Gel Electrophoresis (SDS-PAGE), all samples were diluted into NuPage 4× loading dye (ThermoFisher, NP0008) and boiled at ∼100 °C for 5 min (VWR Scientific Heat Block). SDS-PAGE gels were made in-house with 30% Acrylamide/Bis Solution (37.5:1) (Bio-Rad, 1610158), Tris (Sigma Life Sciences, T1503-1 kg), Sodium Dodecyl Sulfate (SDS; Sigma-Aldrich, L4509-250G), Tetramethylethylenediamine (TEMED; Bio-Rad, 161-0801), and Ammonium persulfate (APS; Bio-Rad, 1610700). 10% SDS-PAGE separating gels contained 10% acrylamide/bis, 370 mM Tris-HCl (pH 8.8), 0.1% SDS, 0.1% TEMED, and 0.03% APS. 4% SDS-PAGE stacking gels contained 4% acrylamide/bis, 120 mM Tris-HCl (pH 6.8), 0.1% SDS, 0.1% TEMED, and 0.1% APS. Gels were subjected to electrophoresis in 1× SDS-Running buffer containing 3 g/L of Tris (pH 8.3), 14.4 g/L of glycine (GoldBio, G-630-1), and 1 g/L of SDS, at 150–200 V for approximately 45 min. All gels included a comparison of protein band molecular weights to the PageRuler Plus Pre-Stained Protein ladder (Thermo Scientific, 26619). The resulting gels were stained overnight in a solution of Coomassie Brilliant Blue G (Sigma-Aldrich, 27815-100G-F) containing 0.125% (w/v) Brilliant Blue G, 50% methanol (PharmCo Greenfield Global, 339000000), 10% acetic acid (EMD Millipore, AX0073-75), and 40% water. Gels were subsequently destained in 50% methanol, 10% acetic acid, and 40% water.

### Transfer Assay with Proteolytic Cleavage

Transfer assays were completed as described above with a 30 min incubation of 4 μM His_6_-LetB-ΔTM with 400 μM host liposomes containing 1% NBD-PE or 1% NBD-PG with 5% DGS-NTA­(Ni^2+^) lipids and*E. coli* extract extruded to 100 nm in 20 mM Tris (pH 8) and 100 mM NaCl. 400 μM acceptor liposomes were then added for a 30 min transfer—these liposomes contained *E. coli* extract extruded to 400 nm in 20 mM Tris (pH 8), 100 mM NaCl, and 0.75 M sucrose. Following the 30 min transfer period, 40 μM chymotrypsin (Sigma, C-4129) was added for a 3-h incubation at room temperature. Donor and acceptor liposomes were then separated by centrifugation at 14,000 × rpm (Eppendorf Centrifuge 5418) for 30 min. Donor-liposome-containing supernatants were removed and acceptor-liposome-containing pellets were resuspended in an appropriate volume of 20 mM Tris (pH 8), and 100 mM NaCl. NBD fluorescence of donor and acceptor solutions was then measured using a Tecan Spark fluorescence/absorbance plate reader (λ_exc_ = 460 nm, λ_em_ = 535 nm). Donor and acceptor NBD-lipid composition was calculated as a percentage of the measured fluorescence signal of each divided by the total measured signal. Negative control experiments were included with no added His_6_-LetB-ΔTM for the subtraction of background NBD-PE or NBD-PG transfer for each replicate.

## Supplementary Material



## #

University of Arizona, Department of Chemistry and Biochemistry; 1306 East University Boulevard, Tucson, Arizona 85721, USA

## Abbrevations

LetB: Lipophilic Envelope-Spanning Tunnel protein B
MCE: Mammalian Cell Entry protein family
C-terminal: Carboxy terminal
N-terminal: Amino terminal
TM: Transmembrane
PE: Phosphatidyl-ethanolamine lipid headgroup
PG: Phosphatidyl-glycerol lipid headgroup
NBD-PE: 7-nitro-2-1,3-benzoxadiazol-4-yl-tagged phosphatidyl-ethanolamine lipids
PG: phosphatidyl-ethanolamine lipids
NBD-PG: 7-nitro-2-1,3-benzoxadiazol-4-yl-tagged phosphatidyl-glycerol lipids
DLS: Dynamic Light Scattering
DGS-NTA­(Ni^2+^): 1,2-dipalmitoyl-*sn*-glycero-3-[(*N*-(5-amino-1-carboxypentyl)­iminodiacetic acid)­succinyl] (nickel salt) lipids
Sodium Dodecyl Sulfate Polyacrylamide Gel Electrophoresis: SDS-PAGE
