# Supplementary material for: Investigation of Lipid Transfer between Model Membranes and the Effect of a Transport Protein on Trafficking
Source: ACS Omega. 2025 Oct 29;10(44):52986–93. doi: 10.1021/acsomega.5c07525 (PMC12612896; doi:10.1021/acsomega.5c07525)
Supplement: Supplementary file 1 [file ao5c07525_si_001.pdf]

Supplemental Materials for:

Title:

Investigation of lipid transfer between model membranes and the effect of a transport protein on trafficking

Authors:

Evelyn W. Cheng[1,2], Megan McDonald[1], Suli Kamholtz-Roberts[1], Virginia Durcan[1], Ashlee M. Plummer-Medeiros[1] *

Affiliations:

[1] Bryn Mawr College, Chemistry Department; 101 N Merion Ave, Bryn Mawr, PA 19010, USA

* Corresponding author; all correspondence: aplummer@brynmawr.edu

[2]Present Address: University of Arizona, Department of Chemistry & Biochemistry; 1306 East University Boulevard, Tucson, Arizona 85721, USA

Supplemental Materials & Methods

Cloning of His$_6$-LetB-ΔTM. pBEL1324 (*i.e.,* LetB(43-877 or ΔTM)-6xHis) was a gift from Gira Bhabha & Damian Ekiert (Addgene #139874; http://n2t.net/addgene:139874; RRID: Addgene_139874)[1]. This plasmid was modified in two steps using Q5 polymerase (New England Biolabs, M0494S) followed by KLD processing (New England Biolabs, M0554S) to 1) add an N-terminal His$_6$ purification tag and 2) remove the C-terminal His$_6$ using the primers below. All PCR reactions were completed in a Bio-Rad PTC Tempo thermocycler. The correct modified sequence was confirmed using Sanger Sequencing (Quintara Biosciences).

Supplemental Table 1) Cloning primers for His$_6$-LetB-ΔTM

	Primer Sequence
1) Add N-terminal His$_6$ tag	Forward: accaccacggcggcggcCAGGACCGGGGTAATAC
	Reverse: gatgatgatggccgccCATGTGAGACCCTCC
2) Remove C-terminal His$_6$	Forward: TAAGGCCAGGCCGGC
	Reverse: GCCTTTGGGAAGCGC

Protein expression. Plasmids encoding for His$_6$-LetB-ΔTM and LetB-ΔTM-His$_6$ were transformed into *E. coli* BL21(DE3) competent cells (Thermoscientific, EC0114) following manufacturer protocols and grown for selection on LB/Agar plates (Lennox LB Broth, Hardy Diagnostics C7652; Agar, Sigma Life Sciences, A1296-500g) with 100 μg/mL ampicillin (Sigma, A-9518). Single colonies were selected and bacterial cultures were scaled up to 500 mL at 37 °C with shaking at 200 rpm (Infors HT Multitron Standard). Optical density at 600 nm was measured using a Thermo Scientific™ NanoDrop™ OneC Microvolume UV-Vis Spectrophotometer and cultures were induced with 1 mM Isopropyl β-D-1-thiogalactopyranoside (IPTG; GoldBio, 12481C25) when OD_{600} reached ~0.6-0.8. Cultures were grown at 37 °C for an additional 4 to 6 hours prior to harvesting via a 30 minute centrifugation at 4000 xg (Thermo Scientific, Sorvall Lynx 4000 centrifuge, F10-4x1000 LEX rotor).

Protein purification. Bacterial pellets were resuspended in lysis buffer containing 20 mM Tris (pH=8.0; Sigma Life Sciences, T1503-1kg), 100 mM NaCl (Sigma-Aldrich, S-3014), 10% glycerol (Sigma, G5516-1L), 0.25 mg/mL DNAse (Sigma-Aldrich, 10104159001), 0.13 mg/mL lysozyme (Sigma-Aldrich, L6876-1g), and cOmplete Mini, EDTA-Free protease inhibitor cocktail (Sigma-Aldrich, 11836170001). Resuspended pellets were lysed via sonication (Qsonica 500 W Model Q500 probe sonicator) on ice using 40% power with a 3s/5s on/off cycling for 5 minutes. Lysate was clarified by centrifugation at 12,000 xg for 45 minutes (Thermo Scientific, Sorvall Lynx 4000 centrifuge, F14-14x50cy rotor). The supernatant was applied to pre-equilibrated HisPur Ni-NTA Superflow Agarose resin (Thermo-Scientific, 25215). Resin was then washed with 10x Column Volumes (CVs) of equilibration buffer (20 mM Tris (pH=8.0), 100 mM NaCl, 10% glycerol), followed by 10x CVs of wash buffer, containing 20 mM Tris, 100 mM NaCl, 10% glycerol, with 20 mM imidazole (Sigma-Aldrich, 12399-500G). Protein was eluted in 5x CVs in 20 mM Tris, 100 mM NaCl, 10% glycerol, with 500 mM imidazole. Eluted protein was concentrated in an Amicon Ultra-15 centrifugation device with a 100 kDa-cutoff (MilliporeSigma, UFC910024) per manufacturer instructions and filtered through a 0.22 μm Costar SpinX Centrifuge Filter (Fisher, 07200386).

Size Exclusion Chromatography**.** All proteins were analyzed by size exclusion chromatographic (SEC) analysis using a GE Akta Pure instrument and a pre-equilibrated Cytiva Superose 6 Increase 10/300 GL column (Millipore Sigma, 29091596) in 20 mM Tris (pH=8.0), 100 mM NaCl, 10% glycerol, operated at 0.4 mL/min. Elution peak volumes were compared to gel-filtration standards with known molecular weights (Bio-Rad, 151-1901).

Protein Concentration Determination. The concentration of protein in SEC fractions of interest was determined using Bradford Assays with Quick Start Bradford 1x Dye (Bio-Rad, 5000205) and Bovine Serum Albumin standard curves (Sigma, A2153-10G) per manufacturer instructions. All measurements are made using 96-well plates (Fisher, 21-377-203) and a Tecan Spark fluorescence/absorbance plate reader (λ= 595 nm).

SDS-PAGE Densitometry. Densitometry analyses (**Supplemental Figure 10**) were completed using the ImageJ built-in density analysis tool[2].

Dynamic Light Scattering. All DLS data were collected on a Wyatt Technology DynaPro NanoStar DLS instrument with a 90 ° detection angle (728-DPN with 2.3.7.3 software version, 661 nm laser). Samples are prepared in disposable microcuvettes (Wyatt Technology, 162960) and the data are collected with a DLS acquisition time of 5 s, read interval of 1 s, and 10 acquisitions are taken with auto-attenuation at 25 °C. Three measurements (*i.e.,* 30 total acquisitions) are averaged. Routine software settings are used, such as viscosity assumptions and fitting with a Rayleigh scattering model.

Lipid transfer assays. Lipid transfer assays in **Supplemental Figure 9** were completed as described in the Methods section with liposomes extruded using a 200 nm Whatman Filter paper (Avanti, 610006).

Supplemental Figures

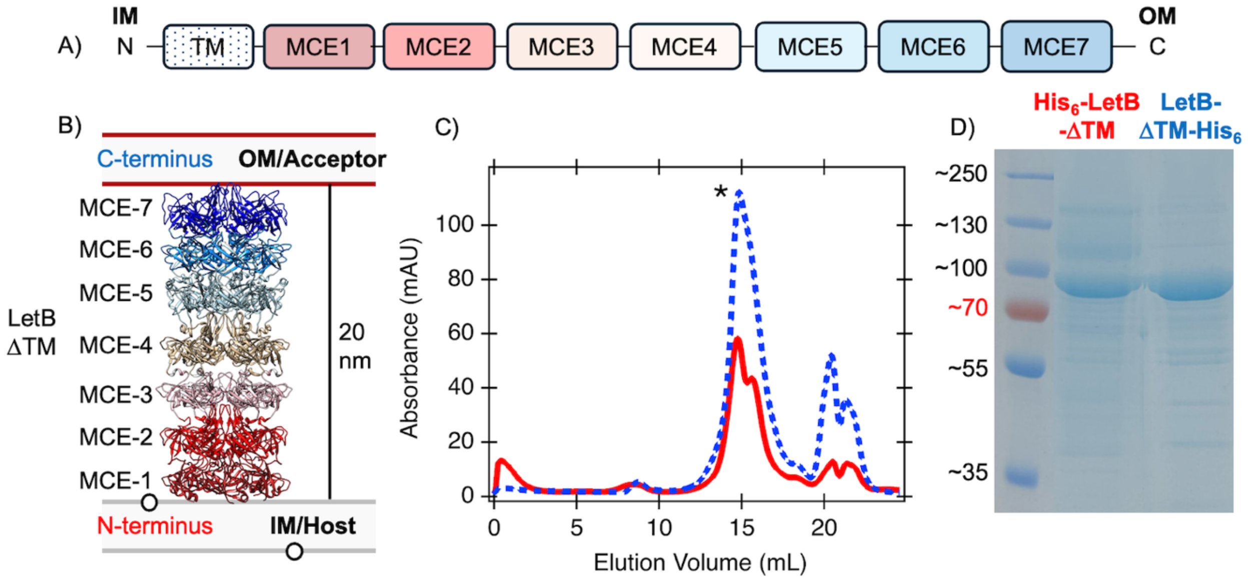

Supplemental Figure 1. The primary sequence of the *E. coli* LetB protein includes an N-terminal transmembrane (TM) helix embedded within the bacterial inner membrane, followed by seven modular Mammalian Cell Entry (MCE) domains (**A**). MCE domains are colored here from N- to C-termini in red to blue. These modular MCE domains physically span the bacterial inner and outer membranes (IM, OM; PDB: 6V0C[1]; **B**). MCE domains are colored here as in (**A**). In our *in vitro* assay, the "host" and "acceptor" vesicles mimic the bacterial IM and OM, respectively. Size-Exclusion Chromatography profiles for His_6-LetB-ΔTM and LetB-ΔTM-His_6 are shown in red and blue, respectively (**C**). Comparison of elution volumes with known standards, including thyroglobulin (670 kDa, 13.2 mL), γ-globulin (158 kDa, 16.3 mL), ovalbumin (44 kDa, 17.4 mL), myoglobin (17 kDa, 18.7 mL), and vitamin B12 (1.4 kDa, 20.3 mL), suggests the expected elution volume of hexameric LetB-ΔTM is 15.4 mL (*). SDS-PAGE analysis of His_6-LetB-ΔTM (red) and LetB-ΔTM-His_6 (blue) shows the expected molecular weight of monomeric LetB-ΔTM is 91 kDa (**D**). UCSF Chimera was used to create this figure [3].

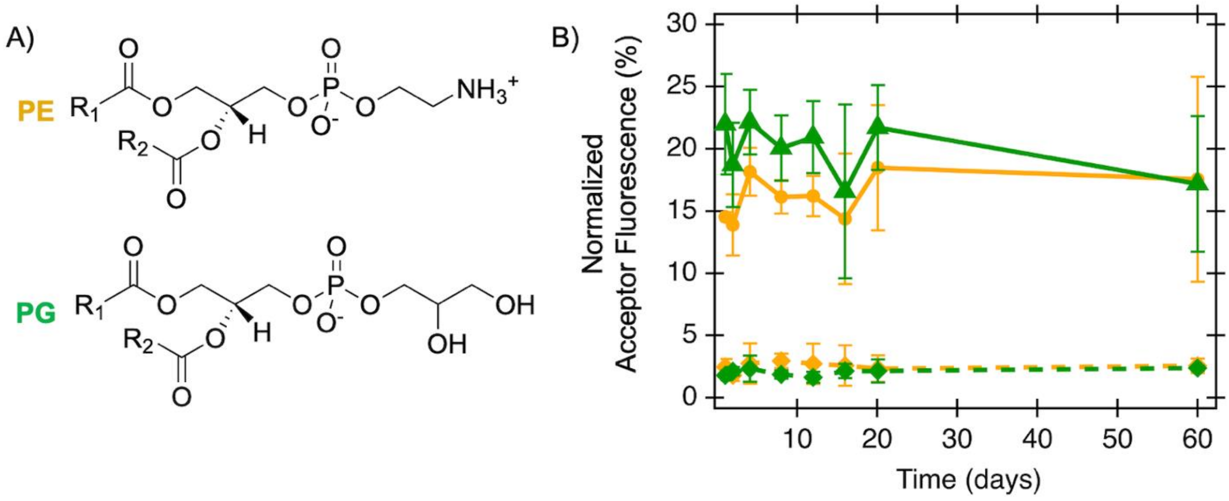

Supplemental Figure 2. The chemical structures of the PE and PG lipid headgroups (**A**). Kinetic experiments monitored transfer over longer (**B**) time scales than shown in **Figure 1B**. Experiments here include 400 μM mixtures of host liposomes with a 100 nm diameter and acceptor liposomes with a 400 nm diameter containing 0.75 M sucrose. NBD-tagged PE (orange) and PG (green) lipids are tested independently. Averages of 3 biological replicates with standard deviations are shown. Dashed lines indicate negative control experiments lacking acceptor liposomes for NBD-lipid transfer.

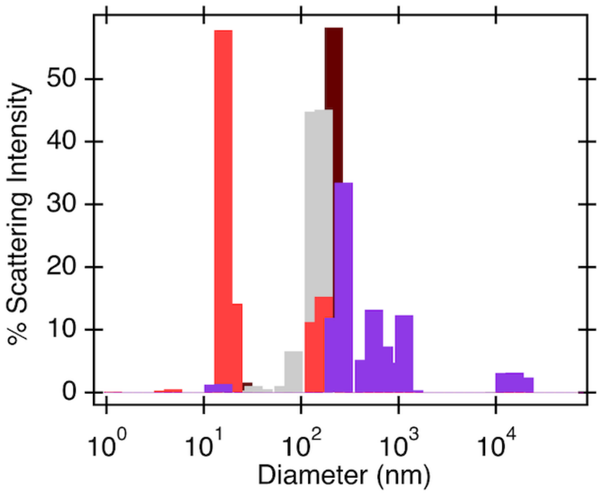

Supplemental Figure 3. Representative DLS scattering intensity profiles of His_6-LetB-ΔTM (red), host and acceptor liposomes (grey, brown, respectively) extruded to diameters of 100 and 400 nm, respectively), and His_6-LetB-ΔTM-anchored host liposomes (purple).

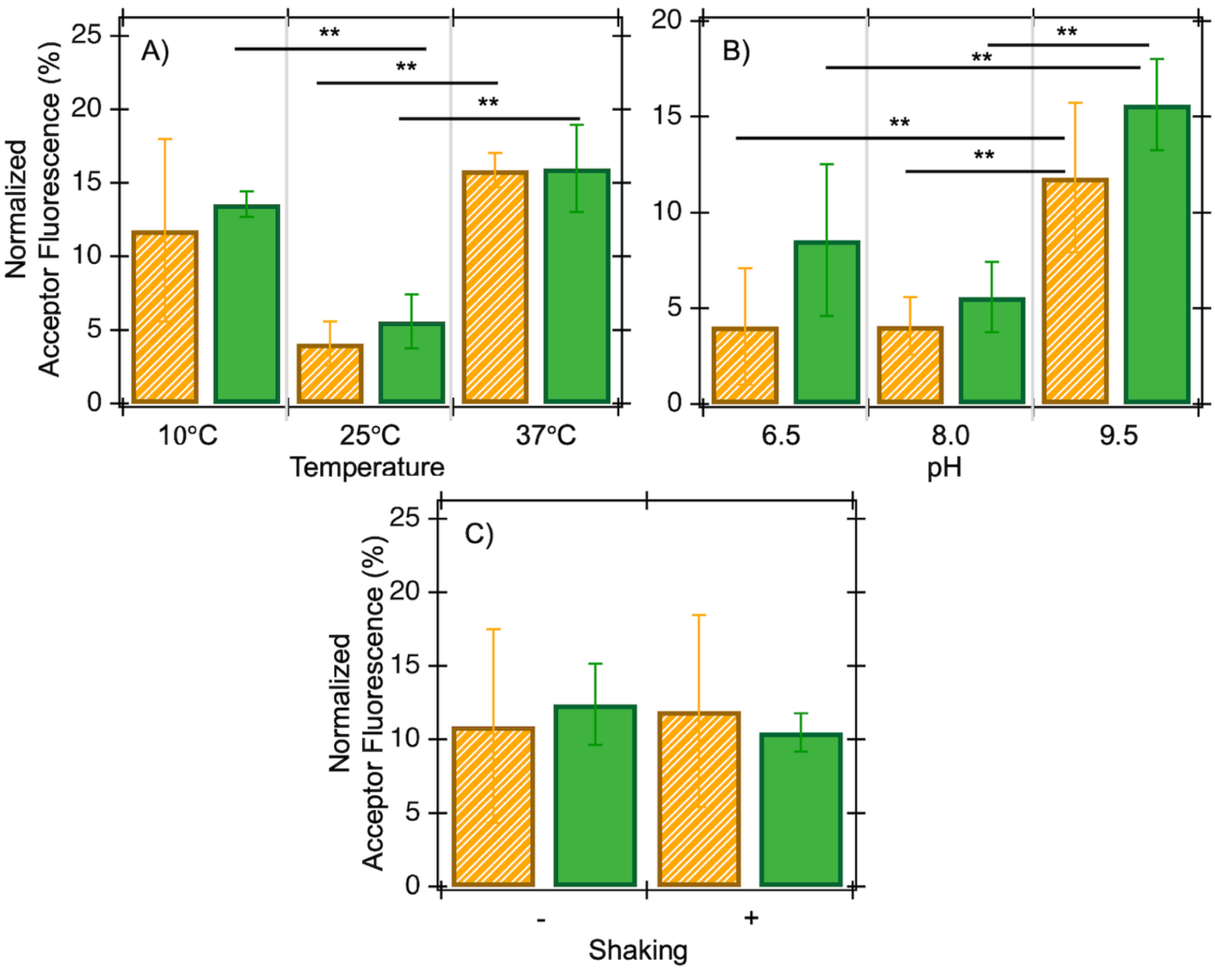

Supplemental Figure 4. Temperature-dependence (**A**), pH-dependence (**B**), and shaking-dependence (**C**) of NBD-tagged lipid transfer. NBD-tagged PE (orange) and PG (green) lipids are tested independently. Experiments here include 1.74 mM mixtures of host liposomes with a 100 nm diameter and acceptor liposomes with a 400 nm diameter containing 0.75 M sucrose separated after 30 minutes. Averages of 3 biological replicates with standard deviations are shown. ** indicates $p < 0.01$ using a 2-tailed t-test assuming equal variance.

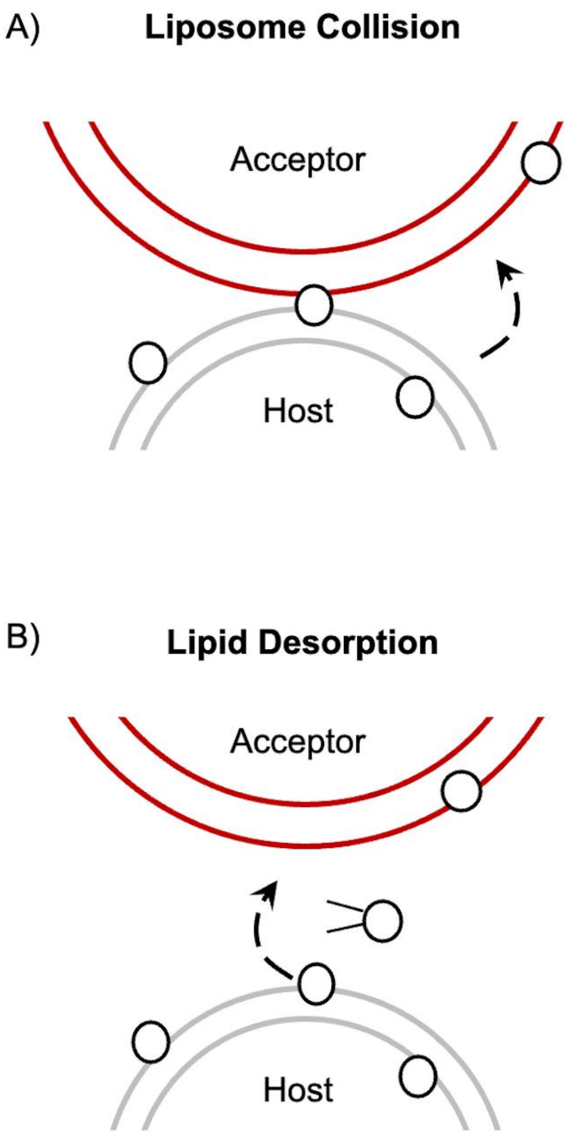

Supplemental Figure 5. Distinct mechanisms for lipid transfer between host (grey) and acceptor (red) liposomes have been reported: lipids (open circles) can be exchanged through liposome collision (**A**) or through individual monomer desorption from host liposomes (**B**).

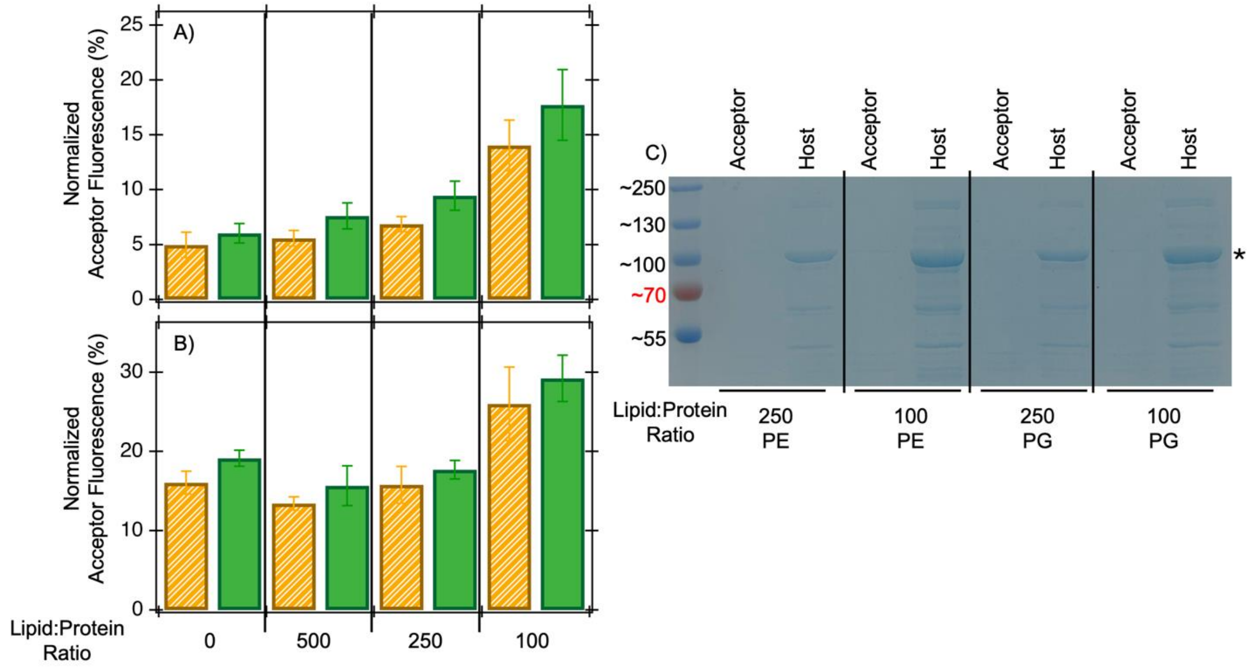

Supplemental Figure 6. LetB–ΔTM-His6 affects the measured transfer of lipids in this *in vitro* assay at measured times of 30 minutes (**A**) and 24 h (**B**). Background spontaneous transfer (*i.e.,* 0 lipid:protein ratio) is shown, along with varying lipid:LetB-ΔTM-His6 molar ratios. NBD-tagged PE (orange) and PG (green) lipids are tested independently. Experiments here include 400 μM mixtures of host liposomes with a 100 nm diameter and acceptor liposomes with a 400 nm diameter containing 0.75 M sucrose separated after 30 minutes. Averages of 3 biological replicates with standard deviations are shown. SDS-PAGE analysis of separated acceptor and host liposomes samples after centrifugation for various tested lipid:LetB-ΔTM ratios (**C**). These data indicate that LetB–ΔTM protein (*) does not cosediment with acceptor liposomes. Importantly, these data do not allow for differentiation of host-liposome bound and soluble, or free, LetB-ΔTM. While LetB may interact with host liposomes under these conditions, it is not facilitating the efficient transfer of NBD-PE or NBD-PG to acceptor liposomes.

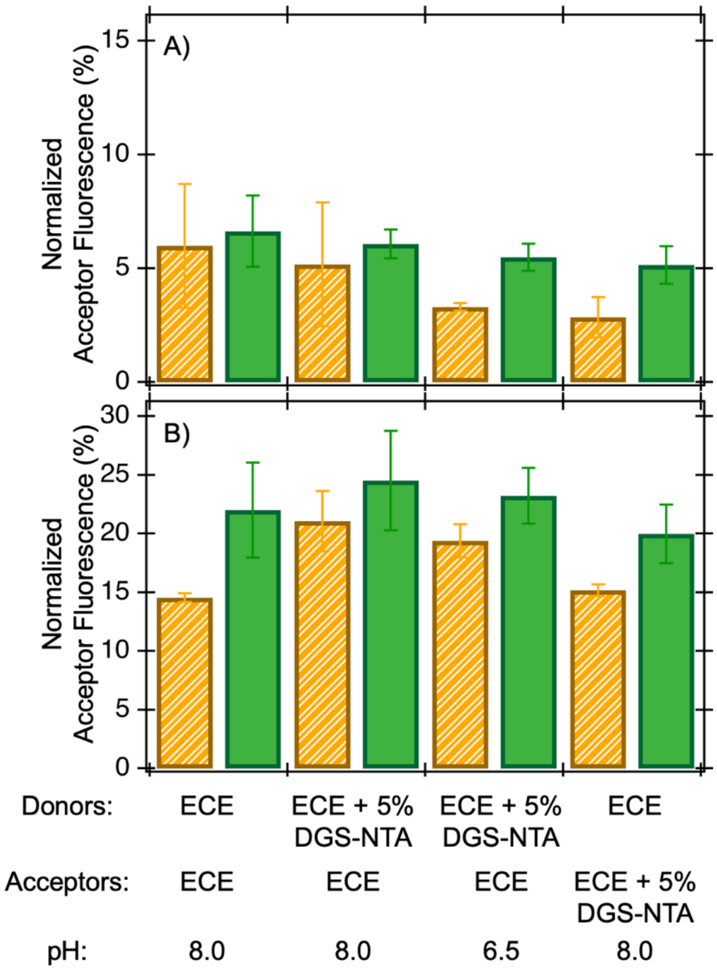

Supplemental Figure 7. Spontaneous lipid transfer with varying donor and acceptor liposome compositions measured at 30 minutes (**A**) and 24 h (**B**). All donors include either 1% NBD-PE or NBD-PG with background lipid composition of E. coli extract (ECE) with or without 5% DGS-NTA(Ni^{+2}) lipids Acceptor liposome composition is also listed and varied to include 5% DGS-NTA(Ni^{+2}) lipids. Experiments here include 400 μM mixtures of host liposomes with a 100 nm diameter and acceptor liposomes with a 400 nm diameter containing 0.75 M sucrose separated after 30 minutes. NBD-tagged PE (orange) and PG (green) lipids are tested independently. Averages of 3 biological replicates with standard deviations are shown.

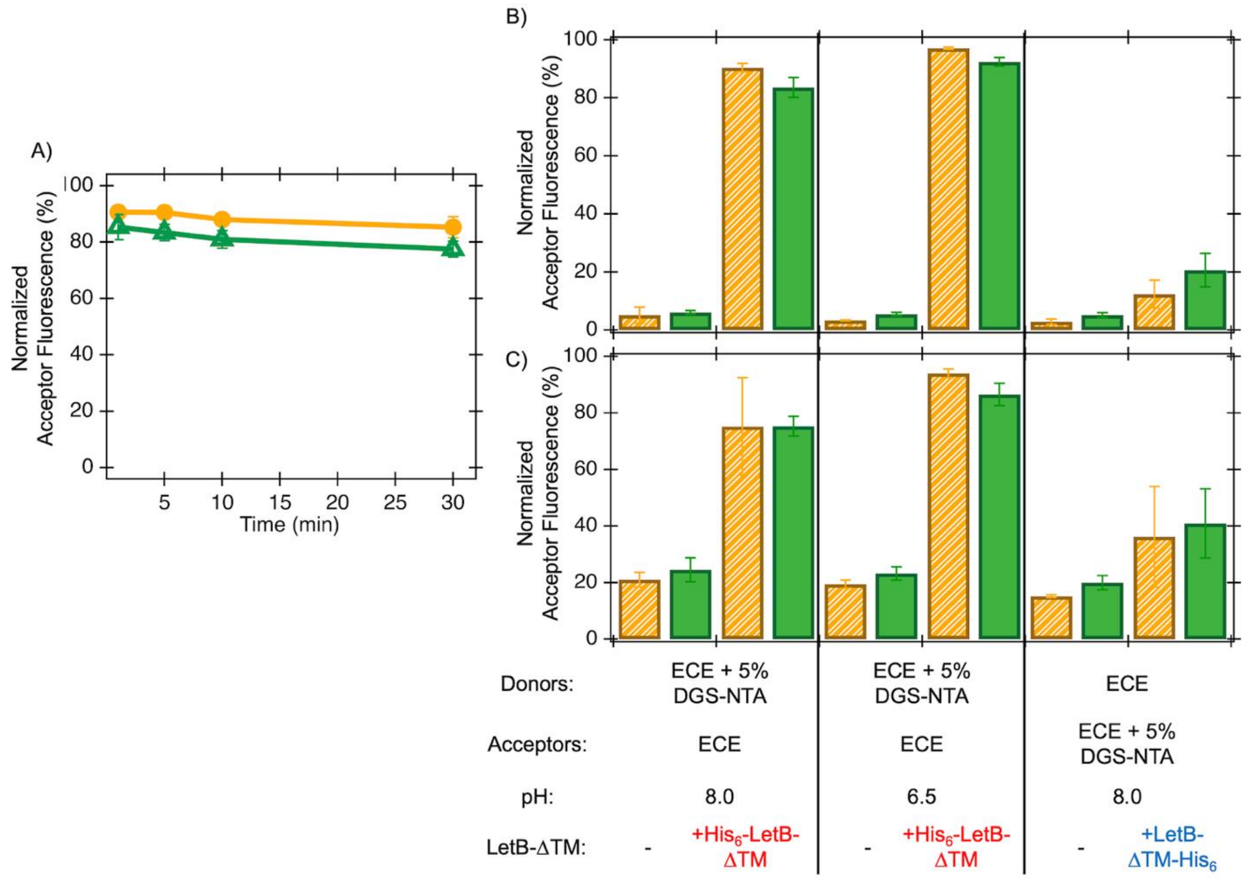

Supplemental Figure 8. Host liposome anchoring of His_6-LetB—ΔTM affects the measured transfer of lipids in this *in vitro* assay at various time points (**A**), at 30 minutes (**B**), and 24 h (**C**). NBD-tagged PE (orange) and PG (green) lipids are tested independently and kinetic experiments monitor the transfer of lipids over a 30-minute time course (**A**). Background spontaneous transfer is subtracted from the LetB-ΔTM-catalyzed amounts here (**A**). Spontaneous transfer (*i.e.,* No LetB-ΔTM) is shown, along with varying combinations of His-tag placement and DGS-NTA(Ni^{+2}) lipid incorporation into donor and acceptor liposomes (**B** & **C**). Experiments here include 400 μM mixtures of host liposomes with a 100 nm diameter and acceptor liposomes with a 400 nm diameter containing 0.75 M sucrose. Averages of 3 biological replicates with standard deviations are shown.

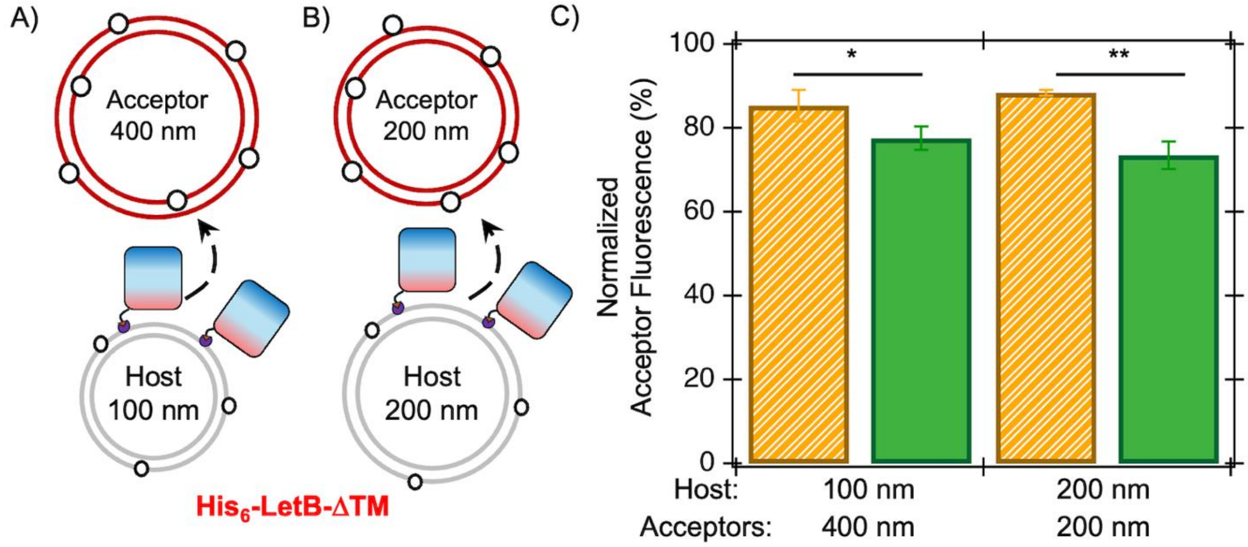

Supplemental Figure 9. The extruded diameters of host and acceptor liposomes minimally affects the observed transfer by His6-LetB-ΔTM. Experiments here include 400 μM mixtures of host liposomes (grey) with either a 100 nm or 200 nm diameter mixed with acceptor liposomes (red) with either a 400 nm or 200 nm diameter, respectively, with 0.75 M sucrose, with 100:1 lipid:LetB-ΔTM ratio, separated after 30 minutes (**A**, **B**). Under both conditions, His6-LetB-ΔTM is anchored to host liposomes through DGS-NTA(Ni^{+2}) lipid (purple) interactions. Similar transfer amounts are observed for both NBD-tagged PE (orange) and PG (green) lipids, which are tested independently (**C**). Background spontaneous transfer is subtracted from the LetB-ΔTM-catalyzed amounts here. Averages of 3 biological replicates with standard deviations are shown. ** indicates $p < 0.01$ using a 2-tailed t-test assuming equal variance; * indicates $p < 0.05$ using a 2-tailed t-test assuming equal variance.

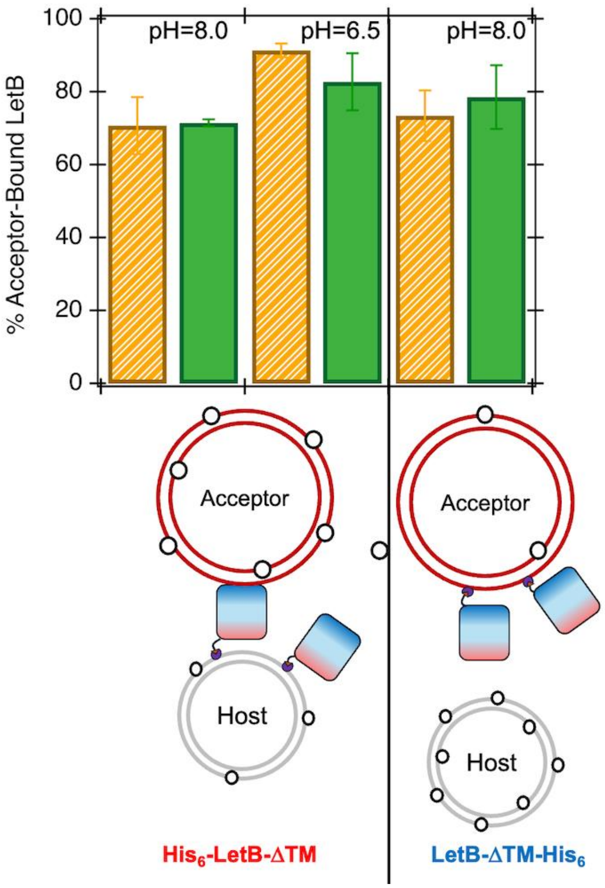

Supplemental Figure 10. Densitometry analyses of experiments shown in **Figure 4A** suggest that both host-liposome (grey) and acceptor-liposome (red) anchored LetB-ΔTM cosediments with acceptor liposomes. Donors with NBD-tagged PE (orange) and PG (green) lipids are tested independently. Importantly, the cosedimentation of LetB-ΔTM-His_6 with acceptor liposomes is mediated through His_6-DGS-NTA(Ni^{+2}) (purple) interactions. Cosedimentation of His_6-LetB-ΔTM with acceptor liposomes cannot occur through His_6-DGS-NTA(Ni^{+2}) interactions, as DGS-NTA(Ni^{+2}) lipids are in the host liposomes in this experiment. Averages of 3 biological replicates with standard deviations are shown.

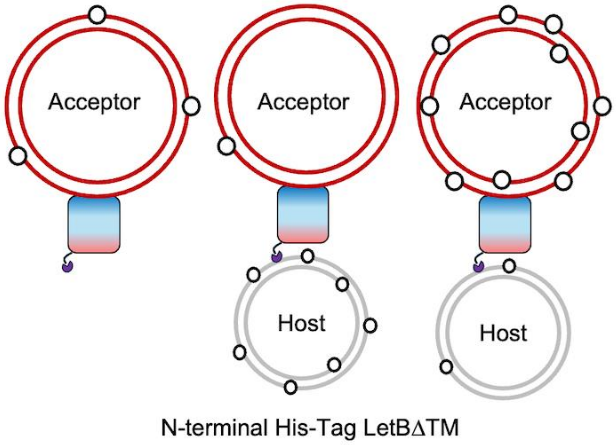

Supplemental Figure 11. Cosedimentation of His_6-LetB-ΔTM (**Figure 4A** & **Supplemental Figure 10**) with acceptor vesicles may arise from several possible molecular arrangements, including dissociation of His_6-LetB-ΔTM from host liposomes and cosedimentation of LetB-ΔTM linked host and acceptor liposomes.

References

1 Isom, G. L., Coudray, N., MacRae, M. R., McManus, C. T., Ekiert, D. C. & Bhabha, G. LetB Structure Reveals a Tunnel for Lipid Transport across the Bacterial Envelope. *Cell* **181**, 653-664 e619 (2020). PMC7335425. https://doi.org:10.1016/j.cell.2020.03.030

2 Schneider, C. A., Rasband, W. S. & Eliceira, K. W. NIH Image to ImageJ: 25 years of image analysis. *Nature Methods* **9**, 671–675 (2012). https://doi.org:doi:10.1038/nmeth.2089

3 Pettersen, E. F., Goddard, T. D., Huang, C. C., Couch, G. S., Greenblatt, D. M., Meng, E. C. & Ferrin, T. E. UCSF Chimera--a visualization system for exploratory research and analysis. *J Comput Chem* **25**, 1605-1612 (2004). https://doi.org:10.1002/jcc.20084
